# Giant Pediatric Supratentorial Tumor: Clinical Feature and Surgical Strategy

**DOI:** 10.3389/fped.2022.870951

**Published:** 2022-04-26

**Authors:** Zhong-Ding Zhang, Huang-Yi Fang, Chen Pang, Yue Yang, Shi-Ze Li, Ling-Li Zhou, Guang-Hui Bai, Han-Song Sheng

**Affiliations:** ^1^Department of Neurosurgery, The Second Affiliated Hospital and Yuying Children's Hospital of Wenzhou Medical University, Wenzhou, China; ^2^The Second School of Medicine, Wenzhou Medical University, Wenzhou, China; ^3^Department of Pathology, The Second Affiliated Hospital and Yuying Children's Hospital of Wenzhou Medical University, Wenzhou, China; ^4^Department of Radiology, The Second Affiliated Hospital and Yuying Children's Hospital of Wenzhou Medical University, Wenzhou, China

**Keywords:** giant pediatric supratentorial tumor, surgery, tumor volume, 3D slicer, gross total resection

## Abstract

**Purpose:**

To analyze the clinical character of giant pediatric supratentorial tumor (GPST) and explore prognostic factors.

**Materials and Methods:**

We analyzed the clinical data comprising of 35 cases of GPST from a single center between January 2015 and December 2020. The tumor volume was measured by 3D slicer software based on preoperative magnetic resonance imaging (MRI). Glasgow Outcome Scale (GOS) was used to evaluate the short-term prognosis.

**Result:**

The tumor volume varied from 27.3 to 632.8 ml (mean volume 129.8 ml/ median volume 82.8 ml). Postoperative histopathological types include ependymoma, pilocytic astrocytoma, choroid plexus papilloma (CPP), craniopharyngioma, primitive neuroectoderm tumor (PNET), choroid plexus carcinoma (CPC), immature teratoma, atypical teratoid rhabdoid tumor (AT/RT), anaplastic astrocytoma, and gangliocytoma. Tumors in children younger than 3 years and tumors located at the hemispheres appeared to be larger than their respective counterparts, though no statistical significance was found. A patient with giant immature teratoma died during the operation because of excessive bleeding. Postoperative complications include cerebrospinal fluid subgaleal collection/effusion, infection, neurological deficits, and seizures. The mean GOS score of patients with GPST in 6 months is 3.43 ± 1.12, and 83% of patients (29/35) showed improvement. Favorable GPST characteristics to indicated better GOS included small tumor (≤100 ml) (*p* = 0.029), low-grade (WHO I-II) (*p* = 0.001), and gross total resection (GTR) (*p* = 0.015). WHO grade was highly correlated with GOS score (correlation coefficient = −0.625, *p* < 0.001). GTR and tumor volume were also correlated (correlation coefficient = −0.428, *p* = 0.010).

**Conclusion:**

The prognosis of GPST is highly correlated with the histopathological type. Smaller tumors are more likely to achieve GTR and might lead to a higher GOS score. Early diagnosis and GTR of the tumor are important for GPST management.

## Introduction

In the United States, brain tumor was the most common malignancy found in children younger than 14 years from 2012 to 2016, with an incidence of 5.55–5.83 cases per 100,000 people (1). Surgical resection is still the main stream treatment with adjuvant radiotherapy and chemotherapy. A giant pediatric intracranial tumor (GPIT), defined as those with a diameter bigger than 5 cm, is especially challenging with high morbidity and mortality (2). Infratentorial tumors and supratentorial tumors have distinctive histological features and surgical strategies, which have been discussed previously including our previous research that analyzed the clinical features of the pediatric giant posterior fossa tumors (3). However, there is a lack of knowledge on their supratentorial counterpart, i.e., giant pediatric supratentorial tumor (GPST).

Pediatric supratentorial brain tumors are often overlooked in the early phase of disease development due to non-specific or even absence of symptoms as signs in children, especially in infants, resulting in the delay of diagnosis (4). Besides, treatment of pediatric brain tumors is very challenging given the unique physiology of children, like rapid development and maturity of various organ systems as well as critical intellectual/mental developments. Radiotherapy and chemotherapy are known to cause endocrine/neurological sequelae and radiotherapy is contraindicated for patients under 3-year-old (5). Therefore, optimization of surgical strategy based on favorable prognostic factors is vital for better care of patients diagnosed with GPST. In this study, we analyzed the clinical characteristics, including tumor volume based on magnetic resonance imaging (MRI), of GPST to explore short-term favorable prognostic factors, which might benefit the management of this disease.

## Materials and Methods

### Patient Selection

We retrospectively analyzed the clinical data of 259 cases of pediatric intracranial tumors treated at the Department of Neurosurgery of the Second Affiliated Hospital of Wenzhou Medical University between January 2015 and December 2020. Inclusion criteria were as follows: (1) The age of the patient is ≤14-year-old. (2) The largest diameter of tumor mass, including cystic components, was ≥5 cm on preoperative MRI. (3) Tumor was primary and with supratentorial location. (4) Patient had no previous intracranial surgery history. (5) Clinical data and follow-up records were complete. As a result, 35 cases were found to be GPST and all received surgical resections. Patients comprised of 22 boys and 13 girls aged from 37 days to 12 years (Table 1).

**Table 1:** Demographic characteristics and sizes of 35 cases of GPST.

**Variable**	**Cases**	**Mean volumes**	***p*-Value**
	**(percentage)**	**(ml)**	
Age groups			
Normal			0.485
<1 year	8 (23%)	173.7 (59.9–632.8)	
1–5 year	14 (40%)	141.0 (40.1–503.7)	
6–10 year	10 (29%)	102.5 (38.2–255.8)	
11–14 year	3 (9%)	60.4 (27.3–94.4)	
Simple			0.202
≤ 3 year	20 (57%)	154.8 (42.6–632.8)	
4–14 year	15 (43%)	98.2 (27.3–255.8)	
Sex			1.000
Males	22 (63%)	141.7 (27.3–632.8)	
Females	13 (37%)	111.8 (40.1–255.8)	
Locations			0.394
Hemisphere	18 (51%)	169.5 (27.3–632.8)	
Lateral ventricle	5 (14%)	111.7 (42.8–229.5)	
Third ventricle	3 (9%)	66.3 (42.6–83.9)	
Sellar region	5 (14%)	84.7 (38.2–230.4)	
Pineal region	1 (3%)	82.8	
Mixed	3 (9%)	84.8 (70.4–107.9)	
Preoperative hydrocephalus			
With	26 (74%)		
Without	9 (26%)		
Symptoms			
Vomiting	25 (71%)		
Headache	17 (49%)		
Gait disorder	15 (43%)		
Muscle weakness	8 (23%)		
Hypersomnia	5 (14%)		
Fever	4 (11%)		
Convulsion	1 (3%)		
Slow response	1 (3%)		

### Data Acquisition

We collected clinical data from the hospital medical record system. Data included the patient's demographic characteristics, MRI and CT images, operation records, and pathology reports. The follow-ups included MRI scanning, neurology examination, and Glasgow Outcome Scale (GOS) evaluation. Every patient was followed for at least 6 months, and death was the endpoint. The tumor volume was measured by 3D slicer software (version 4.10.2, https://www.slicer.org/) by reconstruction of the three-dimensional model based on MRI images.

### Surgical Technique

Surgical management was individualized according to tumor sizes, locations, and features, as well as the physical fitness of the patient. For patients with severe obstructive hydrocephalus, we implanted the Ommaya reservoir or performed external ventricular drainage (EVD) to drain the cerebrospinal fluid (CSF) and lower intracranial pressure before the tumor resection. The surgical goal was to achieve gross total resection (GTR) whenever possible. For tumors with rich vasculatures, we would block the main feeding vessels with bipolar electrocoagulation or an aneurysm clip before formal resection to reduce intraoperative bleeding. Larger craniotomy was preferred for better tumor exposure and manipulations. If the major tumor feeding vessel cannot be found or blocked, segmental resection of the tumor should be avoided due to high risk of massive blood loss. For tumors with cystic components, cystic fluid puncture would first be performed under ultrasound guidance to lower intracranial pressure and reduce the difficulty of surgical resection. However, for highly invasive tumors with abundant blood supply, such as atypical teratoid rhabdoid tumor (AT/RT) and choroid plexus carcinoma (CPC), or tumors adherent to the important structures, subtotal resection (STR) would be performed.

### Statistical Analysis

SPSS 25.0 (IBM Corp., Armonk NY, USA) was used for data analysis. Since the data of tumor volume does not conform to normality, non-parametric tests were employed in its analysis. Two-sample independent Mann–Whitney *U* test was used to compare tumor volume between boys and girls, age groups (“ ≤3 years” group *vs*. “4–14 years” group). Multiple-sample independent Kruskal-Wallis H test was for the comparison of tumor volume in detailed age groups and different locations. Spearman's correlation and Student's *t*-test were employed in prognosis analysis based on GOS. The data were considered statistically significant when *p* < 0.05.

## Result

Demographic data shows that the largest proportion of tumors was from patient age group of 1–5 years (14 cases; 40%). The most common supratentorial site was hemispheres (18/35, 51%); other locations include ventricle (8/35, 23%), sellar region (5/35, 14%), pineal region (1/35, 3%), and mixture (3/35, 9%). The most common symptoms were vomiting (71%), headache (49%), and gait disturbance (43%). Muscle weakness (23%), hypersomnia (14%), fever (11%), convulsion (3%), and slow response (3%) were also seen. The tumor volumes ranged from 27.3 (67 × 40 × 20mm) to 632.8 ml (102 × 124 × 98 mm). The mean volume is 129.8 ± 128.0 ml while the median is 82.8 ml. There was no statistical difference in the tumor volume from different groups of age, sex, and location, despite the mean volume in the younger age group being relatively bigger. But tumors in hemispheres and lateral ventricles (156.95 ± 30.66 ml) were significantly larger than in other locations (51.67 ± 14.92 ml) (*p* = 0.040). Hydrocephalus was found in 74% of patients (Table 1).

Ependymoma was the most common histopathological type in this series, and other types included pilocytic astrocytoma, choroid plexus papilloma (CPP), craniopharyngioma, primitive neuroectoderm tumor (PNET), CPC, immature teratoma, AT/RT, anaplastic astrocytoma, and gangliocytoma. Twenty-nine cases received gross total resection, while five cases only achieved subtotal resection. Sadly, the patient with the biggest tumor in this series (632.8 ml, immature teratoma) died during the operation because of massive bleeding. Postoperative complications occurred in nine cases, including CSF subgaleal collection (six cases), neurological deficits (four cases), pneumonia (one case), central nervous system (CNS) infection (bacterial cerebritis) (one case), and seizure (one case). The postoperative GOS score of patients at 6 months was 3.43 ± 1.12, and most patients got symptomatic and neurological improvement after the surgeries (29/35, 83%). However, the tumor recurred or progressed in six patients (including one case of ependymoma, one case of CPC, two cases of AT/RT, and two cases of PNET), and three of them died within 6 month-time (Table 2).

**Table 2:** Histological types and prognosis of 35 cases of GPST.

**Histological type**	**Cases (percentage)**	**Mean GOS**
Ependymoma(WHO II-III)	10 (29%)	3.50 ± 1.10
Pilocytic astrocytoma (WHO I)	6 (17%)	4.33 ± 0.52
Choroid plexus papilloma (WHO I)	4 (11%)	4.25 (4, 4, 4, 5)
Craniopharyngioma (WHO I)	3 (9%)	3.67 (3, 4, 4)
Primitive neuroectoderm tumor (WHO IV)^*a^	3 (9%)	2.67 (2, 3, 3)
Choroid plexus carcinoma (WHO III)	3 (9%)	1.67 (1, 1, 3)
Immature teratoma (WHO III)	2 (6%)	2.50 (1, 4)
Atypical teratoid rhabdoid tumor (WHO IV)	2 (6%)	3.00 (3, 3)
Anaplastic astrocytoma (WHO III)	1 (3%)	3
Gangliocytoma (WHO I)	1 (3%)	4

*a. These two cases were before WHO removed the “Primitive neuroectoderm tumor” in the new classification system in 2016.

Lower GOS scores were found to be associated with bigger tumor (>100 ml) (*p* = 0.029), high-grade (WHO III-IV) (*p* = 0.001), and subtotal resection (*p* = 0.015). Further, Spearman's correlation analysis also confirmed that GOS was related to tumor volume, tumor grade, and GTR. Among them, the correlation coefficient between the WHO grade of tumor and GOS was −0.625 (*p* ≤ 0.001), indicating that they were highly correlated. Age and sex were not correlated to GOS. Besides, Spearman's correlation between GTR and tumor volume showed that they were also correlated (correlation coefficient =-0.428, *p* = 0.010).

## Discussion

In the past, measurements such as length, width, and height were often used to describe the size of tumors (6). Previous studies have applied “≥5 cm in any diameter” as the standard of giant intracranial tumors for children and analyzed pathological features and surgical outcomes of GPIT or GPST (2, 7). Although this definition is simple and convenient to apply to any GPITs; however, intracranial tumors from different locations vary dramatically in shape and volume. Therefore, it would be inaccurate to measure the volumes of irregular shape tumors through their length, width, and height. Recently, a prospective study used quantitative MRI to calculate cell volume fraction and volume of glioblastoma (8). Another research generates brain tumor volume data in rats through multimodal MRI (9). Therefore, tumor volume measurement based on MRI reconstruction is more accurate and thus would produce more reproducible data. In our routine surgical treatment of pediatric intracranial tumors, we often use 3D slicer software to reconstruct a stereoscopic model of the tumor based on the MRI, which is helpful for surgical planning and displaying it to the family. Therefore, in this case series, we collected the exact volume data of GPST this way.

We found tumors sizes in the younger age group were larger than in older ones: the infant (≤1 year) group had the largest mean tumor volume of 173.7 ml while it was only 60.4 ml in the 11–14-year group. Besides, tumors located in hemispheres and lateral ventricles appeared bigger than those from other locations. It is likely that the difference in tumor size between different age/location groups might contribute to the difference in the onset and manifestations of symptoms. The most common symptoms of this series were vomiting, headache, muscle weakness, and hypersomnia, which were all signs of raised intracranial pressure (ICP). Due to the incomplete closure of the fontanelle, the immaturity of the CNS, and the absence of language development, the signs of increased ICP were usually hard to identify early in infants. Also, patients might be misdiagnosed with other conditions like “gastroenteritis” (10). Besides, symptoms were more likely to be unspecific/non-localized when tumors involved only hemispheres and lateral ventricles when compared to other sites (11). Most intracranial tumors lack specific blood tumor markers, and MRI is the gold standard for radiological diagnosis. Consequently, GPSTs were usually diagnosed only until tumors grow large enough to cause severe symptoms that raise enough suspicion that warrant brain MRI scan.

Ependymoma and pilocytic astrocytoma were the most common histopathology types, followed by CPP, ATRT, PNET, and CPC in our small cohort of patients. Not surprisingly, different histological types would manifest diverse biological characteristics in terms of tumor location, growth rate, and invasion/metastasis tendance, leading to the difference in tumor size at the time of diagnosis (12). However, due to our small cohort, we failed to show any tumor volume differences between different histopathological types. Ependymoma was the most common type (29%, 10/35) in our series, which was in accordance with other reports (2, 13), despite it only accounting for 5.3–10% of all primary CNS tumors (1). Meningioma is a common benign supratentorial tumor found in children (1, 14), but they were rarely reported in GPST studies (2, 15). This might be explained that meningiomas usually lead to early symptoms even when they were small while ependymoma tends to remain asymptomatic until they grow relatively big— so-called “giant” tumors.

We adopted different surgical strategies based on the individual circumstances of each patient and had achieved a low perioperative mortality rate in our serials. It is reported that approximately 60% of pediatric supratentorial tumors are present with hydrocephalus (16, 17). In our series, the percentage was 74% (26/35). Patients with GPST were more likely to have hydrocephalus because the “giant” tumors usually compress the surrounding tissue and block the circulation of cerebrospinal fluid. We routinely implanted Ommaya reservoir or performed EVD to reduce ICP in cases with obstructive hydrocephalus patients before tumor resection. Studies showed that GTR is related to a better prognosis with a higher survival rate and lower recurrences rate (18–20). Our analysis also indicated that patients with GTR or small tumor size (≤100 ml) had higher GOS scores at 6 months. Further analysis suggested that small tumors were more likely to achieve GTR. During the operations, efforts were taken to approach, identify, and resect deeply located tumors. However, it was only feasible to achieve STR in some GPSTs because of the tight adherence to the surrounding structures. In the case of pilocytic astrocytoma at the suprasellar region, we only achieved STR because there were no clear borders between tumor and brain stem tissue. Further resection may be life-threatening or cause catastrophic neurological dysfunction. WHO grade was found to be highly correlated to GOS score. Benign and low-grade tumors were associated with a good prognosis after surgery. However, in high-grade malignant pathological types like AT/RT, CPC, and PENT, tumors not only invaded the surroundings rapidly but also had rich blood supply and unclear tumor margin that render GTR extremely difficult, contributing to the poor prognosis (21). Therefore, treatment of high-grade malignant GPSTs is still challenging. New experimental treatments, including molecular targeted therapy, gene therapy, and autologous stem cell transplantation combined with chemotherapy, have shown preliminary benefits in highly malignant tumors (22–24).

**Figure 1:**
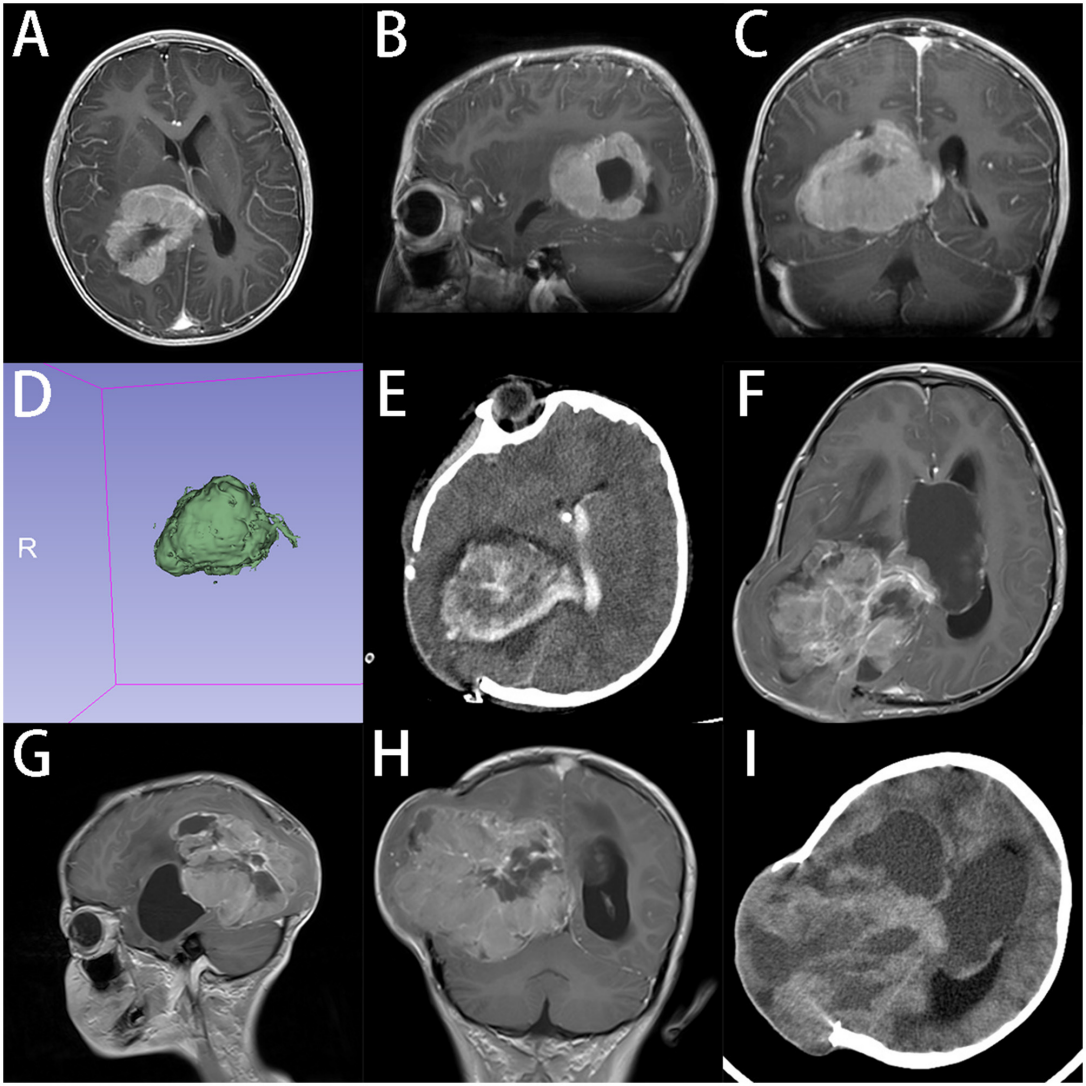
A 7-years-old boy with CPC in lateral ventricles. **(A–C)** Preoperative axial, sagittal and coronal gadolinium-enhanced T1-weighted MRI scan shows an inhomogeneous tumor with partial cystic changes in right lateral ventricle. **(D)** 3D model reconstruction of the tumor, the volume of tumor is 70.4 ml **(E)**. CT shows that tumor was partially removed at 24 h after operation. Guardians reject the advice of secondary surgery after radiation and chemotherapy. **(F–H)** Axial, sagittal, and coronal enhanced T1-weighted MRI scan indicates that tumor recurred at 3 months after operation. **(I)** CT at 4 months after operation, tumor grew further and the condition of patient got worse. Patient died at 5 months after operation.

An early case was a 7-year-old boy who was diagnosed with a choroid plexus tumor in the right lateral ventricle based on the MRI scan, with a volume of 70.4 ml (64 × 46 × 52 mm) (Figure 1). We used the transcortical parieto-occipital approach with tumor location confirmed by intraoperative ultrasound. Because the deep tumor feeding artery was not isolated during the early tumor resection and also the tumor was tightly adhered to the ventricle, the blood loss was massive. Small craniotomy and deep tumor location further restricted our operation field. We had to abandon the initial goal of GTR and converted it to achieve tumor debulking only. The postoperative pathology showed the tumor was CPC instead. As we know, choroid plexus tumors can be divided into CPP, CPC, and atypical choroid plexus papilloma (aCPP), which have quite different prognosis profiles. Most CPCs are not amenable to GTR for their high invasiveness and propensity to metastasize, while GTR can significantly prolong the survival of patients with CPP and aCPP (25, 26). For achieving GTR in CPC, it requires extensive surgical experience and careful preoperative assessment and planning. Preoperative chemotherapy may make resection easier (27). CPC sometimes can be distinguished from CPP based on MRI features like the flowing void effect inside the tumor, but pathological diagnosis is still the gold standard.

**Figure 2:**
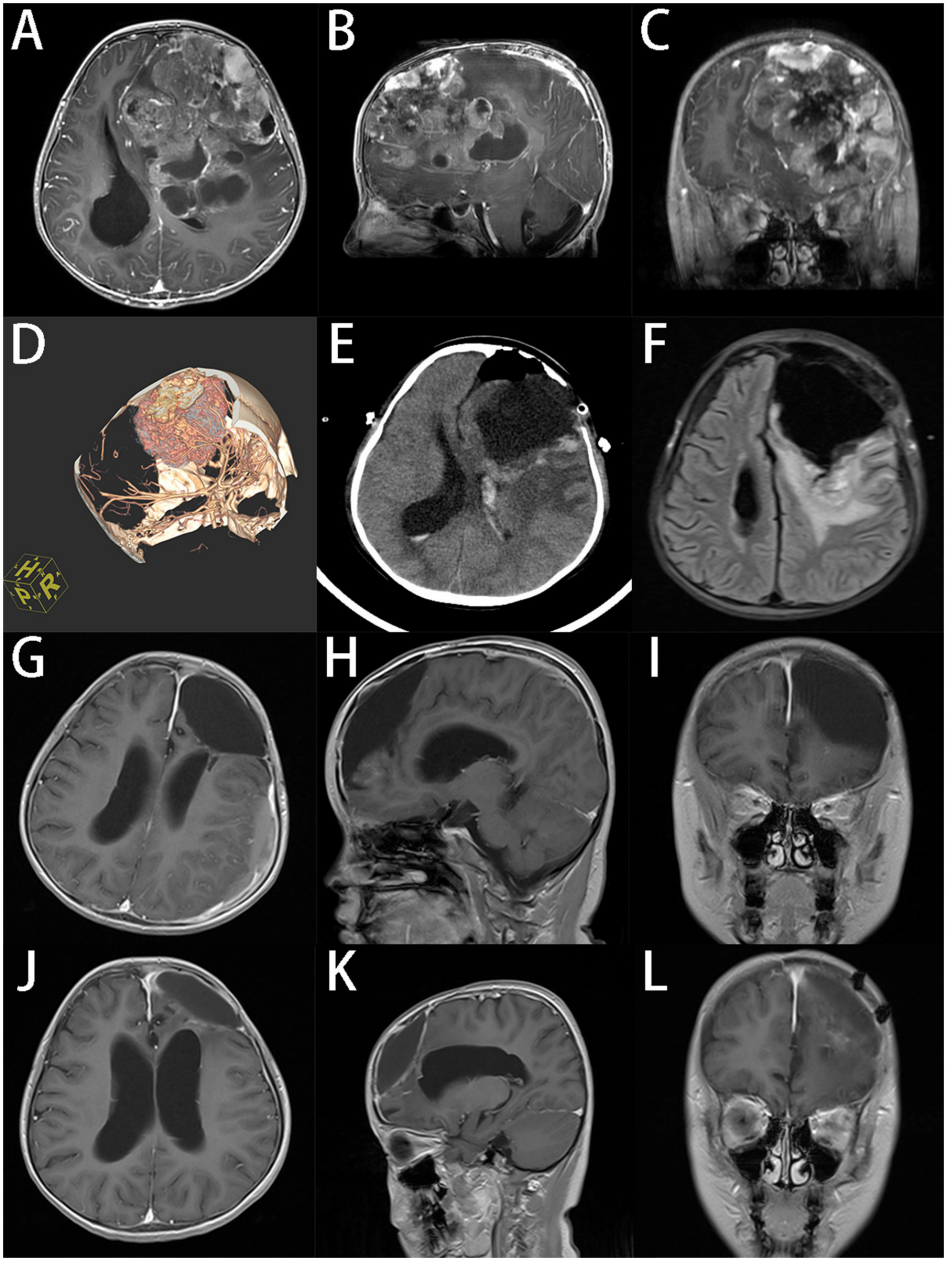
An 8-years-old girl had a giant ependymoma in the left frontal lobe, the tumor volume was 255.8 ml. **(A–C)** Preoperative axial, sagittal, and coronal gadolinium-enhanced T1-weighted MRI scan shows an inhomogeneous enhanced tumor with hemorrhage necrosis and calcification. **(D)** 3D reconstruction of CT angiography. **(E)** Postoperative CT in 24 h after operation. **(F)** Axial MR at 7d after operation. **(G–I)** Axial, sagittal and coronal T1-weighted MRI scan indicates that the tumor was totally removed at 3 months after operation. **(J–L)** Axial, sagittal and coronal T1-weighted MRI scan at 6 months after operation, the residual cavity of tumor reduced.

In this case, we focused on the preoperative evaluation of tumor blood supplies in the treatment of GPSTs. Finding and blocking the feeding vessels before resection can reduce the risk of massive bleeding, which is crucial in the management of tumors with hypervascularity. A large craniotomy is also essential to provide a wider surgical field. However, a larger craniotomy could cause more damage and bleeding, which is associated with more perioperative and postoperative complications (28–30). But we think that expanding the craniotomy area over at least the longest projected diameter of the tumor on the skull is a necessary sacrifice. In a case of an 8-years-old girl with a giant ependymoma in the left frontal lobe, we performed CT angiography before the surgery to confirm the blood supply of the tumor and determined the extent of craniotomy needed based on MRI. During the operation, we clamped the main blood vessels of the tumor and successfully removed the entire tumor with minimal blood loss. The patient recovered rapidly without neurological impairment after surgery. The latest follow-up showed a reduction in residual tumor cavity without sign of recurrence (Figure 2).

Surgery in children under 3-years-old is the most challenging part. Besides, radiation therapy is also contraindicated in this age group. Therefore, tumor resection combined with adjuvant chemotherapy is a common strategy (31). In this series, 20 patients under 3-years-old received the surgery, and 80% of them (16/20) got clinical improvement at 6 months. Four cases had tumor recurrence in 6 months and two cases (Ependymoma and CPC) died in the second and fifth month after surgeries. The prognosis of high-grade malignant tumors is dismal, but, at least to some extent, surgical treatment can prolong survival time and reduce the tumor burden to allow for space for adjuvant treatment. Some surgeons remove GPSTs in the infant with different craniotomy approaches in multistage operations to avoid excessive blood loss (27, 32). However, a staged operation may increase the risk of operation-related complications, and it can be less effective in some highly malignant tumors (7, 33). Our perspective is that GTR is still the goal for most of GPSTs in single stage surgery; reoperation is only reserved for those in whom the first GTR attempt was failed.

Apparently, there are several limitations in our current study. First, the small sample size does not allow for subgroups statistical analysis, e.g., between different histology types. We were also not able to conclude survivals analysis because the follow-up was not long enough. Besides, GOS is not appropriate for infants and toddlers are given their specific developmental stages (34). Actually, there is still a lack of appropriate neurological assessment for this very young age groups. Further study is required to recruit more cases and collect the molecular pathology information in different histology types to better illustrate the clinical significance between clinical features like tumor size and patient prognosis to guide their management.

## Conclusion

The prognosis of GPSTs is highly related to the histopathological type. Early diagnosis and GTR of the tumor are central to the treatment of GPSTs. Although surgery can improve the chance of patient survival, we need to adopt individualized strategies according to the location and pathological features of the tumors to achieve the best possible outcomes. We are more likely to achieve GTR in small tumors and those patients tend to have higher GOS at short-term follow-ups.

## Data Availability Statement

The raw data supporting the conclusions of this article will be made available by the authors, without undue reservation.

## Ethics Statement

The studies involving human participants were reviewed and approved by Ethics Committee of the Second Affiliated Hospital and Yuying Children's Hospital of Wenzhou Medical University. Written informed consent to participate in this study was provided by the participants' legal guardian/next of kin. Written informed consent was obtained from the minor(s)' legal guardian/next of kin for the publication of any potentially identifiable images or data included in this article.

## Author Contributions

Z-DZ: conceptualization, methodology, investigation, formal analysis, writing–original draft, writing–review, and editing. H-YF and CP: conceptualization, methodology, investigation, writing–review, and editing. YY: methodology, investigation, writing–review, and editing. L-LZ and G-HB: methodology, formal analysis, writing–review, and editing. H-SS: conceptualization, methodology, formal analysis, validation, writing–original draft, writing–review, and editing. All authors contributed to the article and approved the submitted version.

## Funding

This study was supported by Health Science and Technology Plan of Zhejiang Province [Grant No. 2021KY794] and Wenzhou Municipal Science and Technology Bureau [Grant No. Y2020419].

## Conflict of Interest

The authors declare that the research was conducted in the absence of any commercial or financial relationships that could be construed as a potential conflict of interest.

## Publisher's Note

All claims expressed in this article are solely those of the authors and do not necessarily represent those of their affiliated organizations, or those of the publisher, the editors and the reviewers. Any product that may be evaluated in this article, or claim that may be made by its manufacturer, is not guaranteed or endorsed by the publisher.
